# A shortened interval between vaccinations with the trivalent inactivated influenza vaccine increases responsiveness in the aged

**DOI:** 10.18632/aging.100852

**Published:** 2015-12-03

**Authors:** Senthil Kannan, Andrew Kossenkov, Raj K. Kurupati, Jason ZQ Xiang, Susan A. Doyle, Kenneth E. Schmader, Louise Schowe, Hildegund C. Ertl

**Affiliations:** ^1^ The Wistar Institute, Philadelphia, PA 19104, USA; ^2^ Gene Therapy and Vaccines Program, University of Pennsylvania School of Medicine, Philadelphia, PA 19104, USA; ^3^ Division of Geriatrics, Department of Medicine, Duke University Medical Center, Durham, NC 27710, USA; ^4^ Geriatric Research, Education, and Clinical Center, Durham VA Medical Center, Durham, NC 27705, USA

**Keywords:** influenza vaccine, antibody responses, gene expression arrays, timing of vaccination

## Abstract

We tested antibody responses to the trivalent inactivated influenza vaccine (TIV) in 34 aged individuals (>65yrs) during the 2012/13 vaccination seasons. Nearly all had been vaccinated the previous year although the time interval between the two vaccine doses differed. One subgroup was re-vaccinated in 2012/13 within 6-9 months of their 2011/12 vaccination, the other received the two doses of vaccine in the typical ~12 month interval. Unexpectedly the sub-cohort with early revaccination exhibited significantly increased response rates and antibody titers to TIV compared to their normally re-vaccinated aged counter parts. Microarray analyses of gene expression in whole blood RNA taken at the day of the 2012/13 re-vaccination revealed statistically significant differences in expression of 754 genes between the individuals with early re-vaccination compared to subjects vaccinated in a normal 12 month interval. These observations suggest that TIV has long-lasting effects on the immune system affecting B cell responses as well as the transcriptome of peripheral blood mononuclear cells and this residual effect may augment vaccination response in patients where the effect of the previous vaccination has not yet diminished.

## INTRODUCTION

Annual influenza vaccination is recommended especially for the very young and the aged and the trivalent inactivated influenza vaccine (TIV) is licensed for use in the general population. The vaccine provides protection against severe influenza through the induction of neutralizing antibodies. Although TIV prevent serious illness in ~ 75% of adults, effectiveness in the aged is still debated [1]. In the 2012/13-influenza season, which started in fall and peaked in January and February, most infections were caused by H3N2 A/Victoria/361/2011 [2]; TIV provided protection to only an estimated 9% of aged vaccine recipients [3]. Whether adjuvanting the vaccine with for example AS03 [4] or using the recently approved high dose vaccine [5] will improve this low level protection of the aged is not known.

In 2011/12 we initiated a study to test antibody responses of aged (≥65 years of age) individuals to the influenza A virus components of TIV. For logistic reasons the first vaccine was given late during the 2011/12 season, between mid-December and the end of March. In the 2012/13-influenza season, we re-vaccinated several of the individuals that were vaccinated later in 2011/2012. Because the 2012/2013 vaccine was given in a timely fashion during fall of 2012, the interval between the 2011/12 and 2012/13 vaccine doses was thus shorter than 12 months (range 6-9 months) for some of these patients. New aged individuals were also recruited and vaccinated on a normal schedule in 2012/13. Antibody responses to the two influenza A virus strains of the TIV were tested from each vaccine recipient at baseline and on days 7 and 14 following vaccination. Unexpectedly, response rates in 2012/13 were markedly enhanced in the aged that received TIV late during the 2011/12 season. Gene expression arrays on whole blood collected at baseline (pre-vaccination) in the 2012/13 season showed significant differences between the two cohorts.

## RESULTS

We tested responses of 34 aged individual in response to the 2012/13 TIV. 15 aged individuals (cohort 1) that had been re-vaccinated within a 6-9 month interval with relation to the 2011/12 TIV, while 18 individuals (cohort 2) had been vaccinated on a regular schedule with 12-13 month intervals between the vaccine doses. One of the aged participants in 2012/13 had never been vaccinated previously and was placed into cohort 2. The majority of the aged (13/15 in cohort 1, 17/19 in cohort 2) reported vaccinations in 2011; there was some bias towards more common annual vaccinations in cohort 2 (cohort 1: 5/15, cohort 2: 15/19; Table 1).

**Table 1:** 

Patient ID	Age	Gender	Vaccine history	Interval between Vaccinations (mos)	Response: H1N1/H3N2
222-003	70	F	2011	10	+/+
222-004	76	M	2011	9	+/+
222-005	88	F	2009, 2011	9	+/−
222-006	72	F	2009, 2011	8	+/+
222-007	79	F	Annually	8	+/+
222-008	84	M	2007, 2009, 2010, 2011	8	+/+
222-009	76	F	2011	9	+/+
222-010	77	F	2011	8	+/+
222-011	74	F	Annually	8	−/−
222-012	77	M	2007, 2011	7	+/+
222-013	66	M	Annually	8	+/+
222-017	74	F	Annually	7	+/+
222-019	68	F	2011	8	+/+
222-020	67	F	2010, 2011	7	+/+
222-021	74	F	Annually	8	+/+
222-031	80	F	Annually	13	−/−
222-032	76	F	Annually	(?)	−/+
222-033	78	M	Annually	11	+/−
222-034	73	M	Annually	(?)	−/−
222-035	76	F	Annually	11	−/+
222-036	69	F	Annually	12	−/+
222-037	75	M	Annually	12	−/−
222-038	77	F	Annually	12	+/+
222-039	73	M	Annually	12	+/−
222-040	85	F	Annually	12	+/+
222-041	82	M	Annually	12	+/−
222-042	77	M	2009, 2010	n.a.	+/+
222-043	81	F	Annually	12	−/+
222-044	80	F	Annually	14	+/+
222-045	82	M	Annually	14	−/−
222-046	80	F	Annually	14	−/−
222-047	77	M	Annually	?	−/−
222-048	72	F	Annually	14	+/+
222-049	75	M	Annually	14	+/−

? – Individuals recall vaccination in 2011 but could not specify the date

n.a. – Not vaccinated in the 2011/12 Flu season

**Figure 1:**
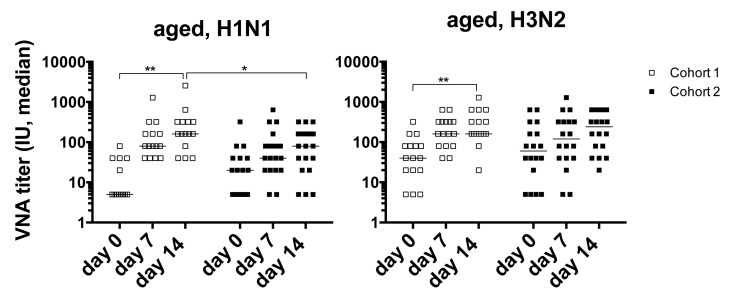
Graphs show VNA titers before and after vaccination in cohorts 1 and 2 Lines with stars above indicate significant differences. H1N1 cohort 1, d0-14: p=0.032; d14 cohort 1 to 2: p=0.011; H3N2 cohort 1 d0 to 14: =0.008.

Sera from all vaccine recipients were tested for virus neutralizing antibodies (VNAs) to the two influenza A virus strains of the vaccine, i.e., H1N1 A/California/7/2009 pdm09-like (H1N1) virus and H3N2 A/Victoria/361/2011 (H3N2) virus before vaccination (day 0) and on days 7 and 14 after the 2012/13 vaccination. Individuals were defined as responsive to a vaccine virus if the VNA titers increased by at least 4 fold over pre-vaccination levels and reached titers of, or above, 1:40 on either day 7 or 14 after vaccination (Figure 1). Unexpectedly, response rates of aged individuals of cohort 1 were higher compared to those of individuals in cohort 2. In cohort 1, 93 and 87% responded to H1N1 and H3N2 virus respectively, while in cohort 2, 63% and 53% responded to H1N1 or H3N2. The latter response rate is closer to what would be expected of an aged population. In addition, only cohort 1 showed significant increases in titers to H1N1 and H3N2 virus after vaccination. Comparisons of the 2 cohorts showed that cohort 1 had significantly higher post-vaccination titers to H1N1 (d7 *p* = 0.04, d14 *p* = 0.01) and significantly higher post-vaccination increases in titers for H1N1 (d7, d14, *p* = 0.02) and for H3N2 (d7, *p* = 0.002). In addition there was a significant inverse correlation in the time interval between the two vaccine doses and absolute post-vaccination antibody titers to H1N1 (d7 *p* = 0.05, d14 *p* = 0.02) and titer increases after vaccination for H1N1 (d7 *p* = 0.01, d14 *p* = 0.004) and H3N2 (d7 *p* = 0.008).

**Figure 2:**
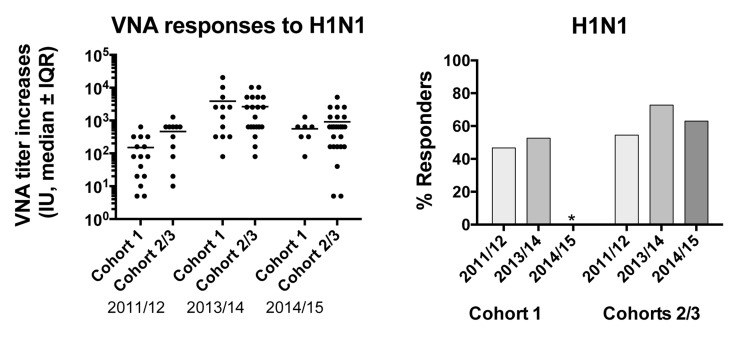
Graph on the left shows increases of VNA titers to H1N1 between d0 and 14 after vaccination for the cohorts tested in different seasons Graph on the right shows responsiveness of the cohorts in the different years.

To ensure that the increased response of cohort 1 was not biased by small numbers of cohort 2 or by other intrinsic differences that allowed cohort 1 to mount better than average responses we tested additional samples collected in the 2011/12, 2013/14 and 2014/15 seasons from individuals of cohort 1 as well of from individuals of cohort 2 and others that enrolled into the study (cohort 3). We compared VNA titer increases to H1N1 at day 14 after TIV over baseline of cohort 1 to those of cohorts 2 and 3, data for the two latter cohorts were combined. In addition we assessed responsiveness by determining the percentage of individuals that mounted a response to H1N1 using the above-described criteria. In 2014/15 too few individuals of cohort 1 enrolled to conduct this comparison. VNA titers of cohort 1 in all other seasons such as 2011/12 and 2013/14 when they were vaccinated on the regular time schedule were indistinguishable from those of other aged individuals. Responsiveness, which on average over the 4 year period was at 59% (excluding cohort 1 2012/13 samples) tended to be lower in the other seasons in cohort 1 than in individuals of the other cohorts. This argues against increased responses of cohort 1 upon a shortened vaccination interval due to some fundamental characteristics that allowed this group of individuals to mount superior antibody responses (Figure 2). In neither cohort, responsiveness in one year was predictive of responsiveness to subsequent vaccinations.

### Gene expression and early revaccination

**Figure 3:**
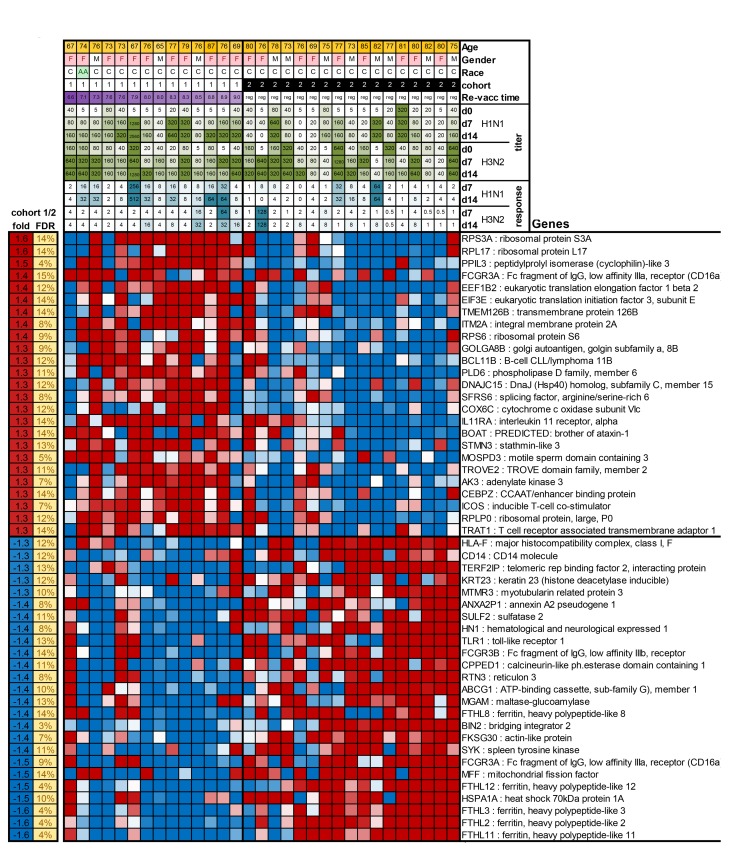
The Figure shows as a heatmap the top 50 genes that are differentially expressed between cohort 1 and 2 In the top of the graph information is provided on patient characteristics, antibody titers before, and on day 7 and 14 after vaccination, and increases in responses after vaccination.

To further understand the basis for the vaccine response differences between cohorts 1 and 2 in the 2012/13 season, we performed gene expression arrays on whole blood collected prior to vaccination on day 0 and compared gene expression profiles between cohorts 1 and 2. We identified a significant differential expression of 786 genes (FDR<15%). A heat map for expression of the top 25 most increased and decreased genes in subjects of cohorts 1 and 2 is shown in Figure 3. A full list of differentially expressed genes is shown in Suppl. Table 1. Several of the transcripts that were increased in cohort 1 are involved in translation (RPL3, RPL10A, RPL38, SFRS6), protein processing and secretion (PPIL3, ITM2A, GOLGA88, SRP72, USP24), metabolism (SC5DL, DENNDAC, ADM2, ATP5H, FAM54A) or lymphocyte stimulation (CD28, ICOS). Genes that were more highly expressed in aged individuals from cohort 2, encode proteins involved in lymphocyte adhesion, mobility and migration (DIP2A, TSPA14, AHAP13, P704P. ILK, LSP1, BIN2), Ca+ flux (ORAI2, ORAI3) and innate immunity (RNF135, MARCO).

Results of Ingenuity Pathway Analysis (Table 2) showed highly significant differences between aged cohorts 1 and 2 in a number of pathways with EIF2 signaling being most significantly increased in cohort 1. Androgen signaling was decreased in cohort 1, which likely reflects the higher proportion of males in the cohort 2 than 1 (44% vs. 20%, respectively). Differences in mTOR and NFAT signaling pathways are suggestive of differences in the activation status of lymphocytes as is further supported by differences in key metabolic pathways, i.e., glycolysis, gluconeo- genesis and cholesterol biosynthesis and by an analysis those involved in apoptosis in cohort 1 (Table 3).

**Table 2:** 

Function	*p*	State	Z	N	↑	↓
**expression of RNA**	4×10^−8^	Inhibited	−2.8	121	43	78
**transcription**	3×10^−7^	Inhibited	−3	111	35	76
**apoptosis**	5×10^−7^	Activated	2.15	138	38	100

p=pvalue of enrichment, State = prediction of function state, Z = z-score of prediction, N=number of genes, ↑ = number of upregulated genes , ↓ = number of downregulated genes

**Table 3:** 

Canonical Pathway	*p*	FDR	State	Z	N	↑	↓
**EIF2 Signaling**	2×10^−6^	0.1%	Activated	3.16	18	15	3
**mTOR Signaling**	0.0001	1.9%		0	15	9	6
**Androgen Signaling**	0.0001	1.9%		−2	11	2	9
**Dopamine Degradation**	0.0003	2.6%			5	0	5
**Glycolysis I**	0.0004	2.6%			5	0	5
**Gluconeogenesis I**	0.0004	2.6%			5	0	5
**Role of NFAT in Regulation of the Immune Response**	0.0005	3.0%		−1.3	12	4	8
**L-DOPA Degradation**	0.0007	3.0%			2	0	2
**Superpathway of Cholesterol Biosynthesis**	0.0007	3.0%			5	3	2
**Protein Ubiquitination Pathway**	0.001	4.1%			16	8	8

p=pvalue of enrichment, FDR = false discovery rate, State = prediction of function state, Z = z-score of prediction, N=number of genes, ↑= number of upregulated genes , ↓= number of downregulated genes

The differentially expressed genes are regulated by several factors that are known to play key roles in lymphocyte activation such as interferon-beta, myc, CD3, RICTOR and HIF-1a as well as by HNF4A and mir133p, which both control metabolism (Table 4). Eleven of the 14 human subjects tested in 2012/2013 from cohort 1 and 11 out of 17 from cohort 2 were revaccinated in 2013/14 and microarray data were gathered from PBMCs at baseline. We found 645 genes differentially expressed at nominal *p*<0.01. Only 25 genes overlapped between the 786 genes found significantly different in the 2012/13 season and the 645 genes that differed in the 2013/14 season with an FDR < 15%. This number is exactly the overlap expected by chance alone, indicating that the differences in year 2012 cannot be explained by endogenous differences between the cohorts.

**Table 4:** 

Regulator	Type	*p*	State	Z	N	↑	↓
**interferon beta-1a**	drug	9×10^−6^		0	13	3	10
**MYCN**	TR	3×10^−5^	Activated	2.18	18	14	4
**miR-103-3p**	microrna	4×10^−5^		−0.1	5	3	2
**CD3**	complex	4×10^−5^		0.71	33	10	23
**HNF4A**	TR	4×10^−5^	Inhibited	−2.9	77	26	51
**RICTOR**	other	6×10^−5^		−1	18	9	9
**HSF2**	TR	7×10^−5^	Inhibited	−2.2	6	0	6

p=pvalue of enrichmentState = prediction of function state, Z = z-score of prediction, N=number of genes, ↑ = number of upregulated genes , ↓ = number of downregulated genes

## DISCUSSION

Influenza virus, a negative stranded RNA virus, has an exceptionally high mutation rate causing constant antigenic drifts and occasional antigenic shifts [6, 7]. Thus although most vaccines induce protection for several years and immunological memory for a lifetime [8], influenza vaccines are given annually to update the strains. Since 2010 the H1N1 component of the vaccine has not changed, while the H3N2 strain has been adjusted. In 2013 H3N2 became the strain that predominated [2]. Overall vaccine efficacy in the 2012/13 influenza season was low at 56% for all age groups. Broken down by age groups, vaccine efficacy was reported to be 27% in the elderly and 67% in children aged 6 months to 17 years (http://www.cdc.gov/flu/pastseasons/1213season.htm).

Especially worrisome was the low efficacy of the H3N2 component in the elderly, which accounted for 50% of all influenza-related hospitalizations [9]. Our report, although based on small cohorts shows that reducing the interval between influenza vaccinations may increase neutralizing antibody response rates in the aged.

Gene expression analysis of whole blood collected at baseline show distinct clustering between individuals that had received the vaccine late in the 2011/12 seasons and the others that had either not been vaccinated (one individual) or been vaccinated in a timely fashion. This finding is already surprising as it indicates that vaccination with TIV has a lasting effect on circulating mononuclear cells.

Previous publications assess gene expression profiles following vaccination. A recent publication assessed the transcriptome of B cells on days 1, 4, 7 and 10 after TIV and identified sets of genes that were associated with robust antibody responses [10]. Another study tested gene expression profiles in whole blood of yellow fever vaccine recipients before and at several time points between days 3-60 after vaccination [11]. In a follow-up study [12] the same group studied early transcriptome changes in purified cell subsets from blood after vaccination with a number of different vaccines including TIV and the life attenuated influenza vaccine [13]. Our study differed in that expression patterns were compared between two aged cohorts that had been vaccinated many months previously. Nevertheless, genes that were differentially expressed after TIV in monocytes, dendritic cell subsets or B cells of their study, overlapped with those that distinguished cohort 1 from 2 in our study suggesting that changes after vaccination are long-lasting. More specifically 90 out of 196 genes in monocytes, 83 out of 712 genes in monocytoid dendritic cells, 79 out of 440 genes in plasmacytoid dendritic cells and 79 out of 722 in B cells were differentially expressed in both studies, which is well above numbers that would be expected by random overlap (10 for monocytes, 37 for monocytoid dendritic cells, 23 for plasmacytoid dendritic cells and 38 for B cells). The over all trend in our study was that transcripts specific for T cells such as CD28, IL-2-inducible T-cell kinase (ITK), T cell receptor associated transmembrane receptor 1 (TRAT1) inducible T cell co-stimulation (ICOS), which plays a major role in the interaction between follicular T helper cells and B cells, and IL-17C, a T cell derived cytokine that regulates innate immune responses were higher in cohort 1 while transcripts specific for cells of the innate immune system were more abundant in cohort 2. The latter included transcripts for NK receptors (LILRB3 and KIR2DL3), proteins specific for monocytes (NCF4, CFP, CD163, TLR-1), neutrophiles (LSP1, IL10RB or macrophages (MARCO).

Other studies that performed microarrays on influenza vaccine recipients reported on positive correlations between responsiveness and transcripts involved in B cell proliferation and Ig production [14]. Another study showed that responsiveness to TIV in young healthy adults was linked to expression of genes involved in antigen processing and intracellular trafficking. Their list of identified genes was distinct from ours [15]. A study that analyzed gene expression profiles in relation to antibody responses to TIV generated gene modules from their whole blood expression data [16]. They showed that a number of modules correlated with antibody responses, such a modules enriched in genes for apoptosis, two modules enriched for genes associated with carbohydrate metabolism or a module for RNA post-trancriptional modifications. Modules that in their study predicted responsiveness to TIV were not linked to differences in gene expression in our study.

Overall our study shows superior responses rates in aged individuals that received the annual influenza vaccine in a less than 12-month interval. Individuals of the same group vaccinated on a regular schedule in previous or subsequent years show responses similar to those of other aged human subjects. Gene expression arrays showed that shorter interval between vaccine dose decreased transcripts indicative of innate responses and increased transcripts linked to T cell responses as well as transcripts encoding genes involved in translation.

## METHODS

### Virus strains

The two influenza A vaccine strains of the 2012/13 seasonal influenza vaccine, A/California/7/2009 (H1N1)pdm09-like virus and A/Victoria/361/2011 (H3N2)-like virus were obtained from the Center for Disease Control, Atlanta, Georgia. Viruses were expanded in 10 day-old specific pathogen-free embryonated eggs. Cleared allantoic fluids was purified by fractionation over 10-55% sucrose density gradients at 25,000rpm for 2 hrs. Mean tissue culture infective dose (TCID_50_) was determined by titration on Madin-Darby Canine Kidney (MDCK) cells.

### Human subjects

Blood was collected in the Duke Clinical Research Unit (DCRU) after informed consent from community dwelling persons in the Durham-Raleigh-Chapel Hill area of North Carolina. Individuals were > 65 years of age. Subjects with underlying diseases or therapies that affect the immune system, acute febrile infections, as well as subjects, which were bed-ridden or homebound or were unlikely to adhere to protocol follow-up were not enrolled. Subjects with contraindication for influenza vaccination such as anaphylactic hypersensitivity to eggs or to other components of the influenza vaccine, and moderate or severe acute illness with or without fever, and Guillain-Barre Syndrome within 6 weeks following a previous dose of influenza vaccine were not enrolled. Demographic data and medical history including medical diagnoses, medications, vaccination to influenza and other infectious diseases, and history of influenza or influenza-like diseases during the last 5 years were recorded. Subjects were bled and vaccinated with TIV. Subjects were bled again on days 7 and 14 following injection of TIV.

### Blood and serum samples

Blood was collected and serum was isolated and heat-inactivated by a 30 min incubation at 56°C prior to testing. The blood samples for the gene expression analyses were collected into PaxGene tubes to immediately stabilize RNA for analysis [17]. Samples were shipped overnight from the point of collection in the DCRU to The Wistar Institute in Philadelphia. Serum was isolated and frozen at −20°C till further use. PAXgene tubes were stored at −80°C until RNA extraction. RNA was extracted using the PAXgene Blood RNA Kit IVD for isolation and purification of intracellular RNA from blood stabilized in PAXgene Blood RNA Tubes according to the manufacturers directions. RNA integrity was assessed using a bioanalyzer and only samples with an RNA integrity (RIN) # of >7.5 were processed for arrays. A constant amount (400 ng) of total RNA was amplified, as recommended by Illumina and hybridized to the Illumina H12-v4 human whole genome bead arrays.

### Micro-neutralization assay

Two-fold serially diluted (1:20 to 1:10240) heat-inactivated human sera were tested for neutralizing antibodies to influenza A virus strains by micro-neutralization assays. Equal volume of 100TCID_50_ per well of the titrated virus was added to the diluted serum in 96 well plates and incubated at 37°C. After 1hr, serum-virus mixtures were added to MDCK cells that had been washed twice with serum-free Dulbecco’s Modified Eagles Medium (DMEM). The cells were incubated for 2 hrs at 37°C with 5% CO_2_. The cells were washed and re-incubated with DMEM supplemented with L -1-Tosylamide-2-phenylethyl chloromethyl ketone (TPCK) trypsin for 3 days. CPEs were scored under a microscope. Neutralization titers were defined as the dilution of the serum that resulted in 50% inhibition of CPE formation.

### Statistical analyses

Post-vaccination titers and post-vaccination increases in titers for H1N1 and H3N2 were compared between two groups using Mann-Whitney non-parametric test. Correlation analysis with time interval between the two vaccine doses was done using Spearman correlation. Results that passed *p*<0.05 threshold were called significant.

### Microarray data analysis

All arrays were processed in the Wistar Institute Genomics Facility. Illumina GenomeStudio software was used to export expression levels and detection p-values for each probe of each sample. Signal intensity data was quantile normalized and genes that showed insignificant detection p-value (*p*>0.05) in all samples were removed from further analysis. Expression level comparisons between two groups were done using two sample t-test and correction for multiple testing to estimate False Discovery Rate (FDR) was done with Storey et al. procedure [18] and FDR<15% was used as a significance threshold. Gene set enrichment analysis for biological functions, canonical pathways and upstream regulators was done using QIAGEN's Ingenuity® Pathway Analysis software (IPA®, QIAGEN Redwood City, http://www.qiagen.com/ingenuity) and significance of enrichment was defined at FDR<5% for pathways, *p*-value<10^−6^ for functions and *p*-value<10^−5^ for upstream regulators.

## SUPPLEMENTAL MATERIAL

Please browse the Full Text version to see the Supplemental Table of this manuscript.
